# Retraction: Functional Study of Ectodysplasin-A Mutations Causing Non-Syndromic Tooth Agenesis

**DOI:** 10.1371/journal.pone.0351787

**Published:** 2026-06-17

**Authors:** The PLOS One Editors

After this article [1] was published, concerns were raised regarding Figs 1 and 2. Specifically,

- In Fig 1A, the bands in the H252L and Y343C lanes appear similar to each other, and there appears to be a vertical discontinuity between these lanes.
- In Fig 1C:
  - The bands in the WT and A259E lanes appear similar to one another.
  - The bands in the H252L and Y343C lanes appear similar to one another, and there appears to be a vertical discontinuity between these lanes.
- In Fig 2A, the bands in the H252L and Y343C lanes appear similar to each other.
- The image background details do not appear visible for many of the western blot panels in [1].

The corresponding author stated that the Y343C band in Fig 2A was included to verify the intracellular expression of EDA2 in the corresponding cells, was obtained from a separate membrane and was not used for quantitative comparison. The underlying images provided did not resolve the above concerns and raised further concerns with the results presented in Figs 1 and 2.

In light of the above concerns that question the reliability and integrity of the reported results and conclusions, the *PLOS One* Editors retract this article.

MLS agreed with retraction and apologizes for the issues with the published article. WS, YW, YL, HZ, DH, and HF did not agree with retraction and stand by the article’s findings. GZ either did not respond directly or could not be reached.
